# *DCDC2, KIAA0319* and *CMIP* Are Associated with Reading-Related Traits

**DOI:** 10.1016/j.biopsych.2011.02.005

**Published:** 2011-08-01

**Authors:** Tom S. Scerri, Andrew P. Morris, Lyn-Louise Buckingham, Dianne F. Newbury, Laura L. Miller, Anthony P. Monaco, Dorothy V.M. Bishop, Silvia Paracchini

**Affiliations:** a Wellcome Trust Centre for Human Genetics, University of Oxford, UK; b School of Social and Community Medicine, University of Bristol, UK; c Department of Experimental Psychology, University of Oxford, UK

**Keywords:** ALSPAC, association study, dyslexia, language, reading abilities, specific language impairment (SLI)

## Abstract

**Background:**

Several susceptibility genes have been proposed for dyslexia (reading disability; RD) and specific language impairment (SLI). RD and SLI show comorbidity, but it is unclear whether a common genetic component is shared.

**Methods:**

We have investigated whether candidate genes for RD and SLI affect specific cognitive traits or have broad effect on cognition. We have analyzed common risk variants within RD (*MRPL19/C2ORF3*, *KIAA0319,* and *DCDC2*) and language impairment (*CMIP* and *ATP2C2*) candidate loci in the Avon Longitudinal Study of Parents and Children cohort (*n* = 3725), representing children born in southwest England in the early 1990s.

**Results:**

We detected associations between reading skills and *KIAA0319*, *DCDC2,* and *CMIP*. We show that *DCDC2* is specifically associated with RD, whereas variants in *CMIP* and *KIAA0319* are associated with reading skills across the ability range. The strongest associations were restricted to single-word reading and spelling measures, suggesting that these genes do not extend their effect to other reading and language-related skills. Inclusion of individuals with comorbidity tends to strengthen these associations. Our data do not support *MRPL19/C2ORF3* as a locus involved in reading abilities nor *CMIP/ATP2C2* as genes regulating language skills.

**Conclusions:**

We provide further support for the role of *KIAA0319* and *DCDC2* in contributing to reading abilities and novel evidence that the language-disorder candidate gene *CMIP* is also implicated in reading processes. Additionally, we present novel data to evaluate the prevalence and comorbidity of RD and SLI, and we recommend not excluding individuals with comorbid RD and SLI when designing genetic association studies for RD.

Dyslexia (or reading disability, RD) and SLI are common childhood disorders. RD is a specific deficit in learning to read, whereas SLI refers to an impairment in the acquisition of oral language (1). The biological cause of RD and SLI remains poorly understood, but it is clear that their manifestation is the result of multiple interacting factors, many of which have a genetic origin. Family studies have reported that, for both disorders, first-degree relatives of affected individuals also have a 30% to 50% chance of being affected, whereas the general population prevalence is approximately 5% (2,3). Comorbidity between RD and SLI has been consistently reported. Estimates indicate that 43% of children with SLI are later diagnosed with RD (4), and up to 55% of children with RD meet criteria for SLI (5). These figures have led to the hypothesis that SLI and RD may be manifestations of the same underlying deficit or may share etiologic factors, such as genetic determinants (1). Both SLI and RD show increased comorbidity with attention-deficit/hyperactivity disorder (ADHD), another common neurodevelopmental disorder, affecting 3% to 5% children (6). It is estimated that 25% to 40% of children with RD manifest symptoms of ADHD as well (7), and children with language disorder are at higher risk of developing ADHD (8).

Several genes have been proposed as susceptibility candidates for either RD or language-related skills and have been extensively reviewed (9−11). The RD candidates include the *MRPL19/C2ORF3* locus, *ROBO1*, *KIAA0319*, *DCDC2,* and *DYX1C1*. With the exception of *ROBO1* (12), these genes are supported by genetic associations with common single nucleotide polymorphisms (SNPs).

*DYX1C1* was the first RD candidate to be identified following breakpoint mapping of a translocation cosegregating with RD (13). Association analysis in a cohort with RD implicated two putative coding variants: the -3A (rs3743205) and the 1249T (rs57809907) variants. A large number of replication studies have not reached consensus in supporting *DYX1C1* RD susceptibility variants (14−22).

More consistent observations have been reported for the *KIAA0319* and *DCDC2* genes located at the chromosome 6 locus. Most of the associations with *KIAA0319* cluster around the 5′ end of this gene and generally show the same allelic trend across independent studies (23−27). Functional studies showed that one particular RD-risk haplotype, effectively tagged by the minor allele of the rs2143340 SNP, is associated with reduced expression of *KIAA0319* (28). This haplotype also harbors the minor allele of rs9461045, creating a binding site for a nuclear protein, which could explain the reduced gene expression and provides a functional mechanism underlying the genetic associations (29). Other studies did not find associations within *KIAA0319* but identified the nearby *DCDC2* gene as an RD candidate (30,31). Replication studies in samples selected for RD provided further support for *DCDC2* but with modest associations (14,23,32,33). Rare variants located between these two genes have been found to be associated with speech perception in children with dyslexia (34).

The *MRPL19* and *C2ORF3* genes, which appear to be coregulated, are supported by single intergenic SNPs and overlapping haplotypes yielding significant associations in two independent samples of Finnish and German origin (35).

The candidate genes for language include *CMIP*, *ATP2C2,* and *CNTNAP2*. *CMIP* and *ATP2C2* have been associated with nonword repetition, which is regarded as a measure of phonologic short-term memory, in samples of individuals with language impairment (36). Both genes were identified following high-density mapping at the chromosome 16 locus for SLI (37). The associations, originally identified in a cohort of individuals with SLI, were also seen in a subgroup of individuals selected on the basis of low language skills from the Avon Longitudinal Study of Parents and Children (ALSPAC) cohort (38), albeit with an opposite direction of trend for *CMIP*. Instead, no associations were detected with language traits in the entire ALSPAC cohort, which is representative of the general population. This suggests that the two genes have an effect on nonword repetition on a background of language impairment.

*CNTNAP2* genetic variants were found to be associated with language-related phenotypes and a task of verbal short-term memory in the same language-impaired cohort used for *CMIP* and *ATP2C2* (39). *CNTNAP2* is a target of FOXP2 (39), which is implicated in severe and rare forms of language impairment (40).

The use of epidemiologic cohorts has proved to be a valid approach to investigate further genetic associations with some of these genes. The *KIAA0319* RD-associated haplotype (26) was significantly associated with reading skills in both ALSPAC (41) and in a twin-based Australian sample (42), but with an opposite trend in the latter. The same Australian sample was also used to investigate *DCDC2* (43) and *DYX1C1* (44).

Phenotype definition is a key component when investigating the genetics of language and reading disorders. Tests of single-word reading are the most commonly used measures in genetic association studies of RD (45). Nonword repetition is a good marker for heritable SLI (46). However, an important issue is how far language problems should be identified solely by psychometric tests, which may miss key features of communication difficulties. Parental reports can be highly effective in identifying heritable communication problems (47,48), but they typically identify a different subset of children than those identified on direct language testing (49).

Bishop and Snowling (50) noted that RD and SLI were for a long time regarded as distinct disorders but in recent years have been reconceptualized as points on a continuum. This is an oversimplification, because different components of language and reading skills can fractionate, but it is possible that the same genetic components could contribute to both disorders and explain, at least partially, the observed comorbidity. Previous studies exploring the role of shared genes in contributing to both RD and SLI indicate *KIAA0319* as a possible common risk factor supported by associations with language-related measures in samples selected for language impairment (51,52). One of these studies also showed that *CMIP* was associated with both reading and language-related measures in the same sample selected for language impairment (52). No association with language measures was reported for an investigation of *DCDC2* and *DYX1C1* in a sample of families ascertained for dyslexia (53). A genetic overlap has been suggested for RD and ADHD by a linkage study (54), and *DCDC2* has been suggested to contribute to both RD and ADHD (55). *ATP2C2* has also been found associated with ADHD (56).

Here, we investigated in the ALSPAC cohort genetic associations reported in the literature from samples selected for either RD or SLI. We conducted association analysis to 1) replicate associations with reading and nonword repetition measures, 2) dissect the phenotypic components of such associations by testing different but related quantitative phenotypes to pinpoint the underlying cognitive deficit(s), and 3) test for pleiotropic effects across reading- and language-related measures. We identified association between reading abilities and the *DCDC2*, *KIAA0319,* and *CMIP* genes and have shown that these associations follow different patterns; whereas *DCDC2* is associated more specifically with dyslexia, *CMIP* and *KIAA0319* are associated with reading abilities in the normal range. In addition, we show that these genes have a specific effect on a test of single-word reading rather than a more generalized impact. Lastly, we have evaluated the effect of individuals with comorbid RD and SLI in association analysis. Our results suggest that the inclusion of these individuals may increase power in genetic association studies for dyslexia.

## Methods and Materials

We genotyped the ALSPAC children cohort (*n* ∼11,000) using either Sequenom iPLEX assays (San Diego, California) or the KBiosciences (Herts, United Kingdom) service using their in-house technology. Nineteen SNPs passed the quality control criteria of a call rate greater than 90%, error rate less than 1.5% (estimate derived from approximately 3% of samples blindly distributed in duplicates), minor allele frequency greater than .05, and genotype frequencies in Hardy−Weinberg equilibrium (*p* > .05). We had good quality data for SNPs within *MRPL19/C2ORF3, KIAA0319, DCDC2*, *ATP2C2,* and *CMIP* but not for *DYX1C1* and *CNTNAP2*, which therefore were not included in this analysis.

Both quantitative and case−control analyses were performed within PLINK (57) testing for additive effects.

**Figure 1:**
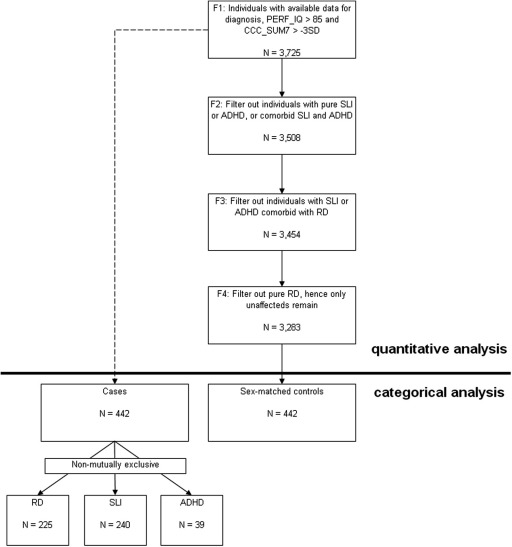
Diagram illustrating how phenotypic subgroups were identified. The subgroups above the black horizontal lines were used for quantitative analysis while the ones below were used for case-control analysis. The extent of co-morbidity (hence the non-mutually exclusive definition of cases), can be seen in Figure 2. ADHD, attention-deficit/hyperactivity disorder; CCC_SUM7, sum of first seven scales from the Children's Communication Checklist; PERF_IQ, performance IQ; RD, reading disability; SLI, specific language impairment.

We based our initial analysis on the F1 sample (Figure 1; Methods in Supplement 1), which includes all available individuals after filtering for missing data, ethnicity, IQ, and autistic traits. Individuals were then assigned to the groups of RD, SLI, ADHD, any of the four comorbid combinations of these three disorders, or unaffected (Figure 1, Table 1). See Methods in Supplement 1 for full description of sample subgroups.

## Results

### Observed Disorder Prevalence

**Figure 2:**
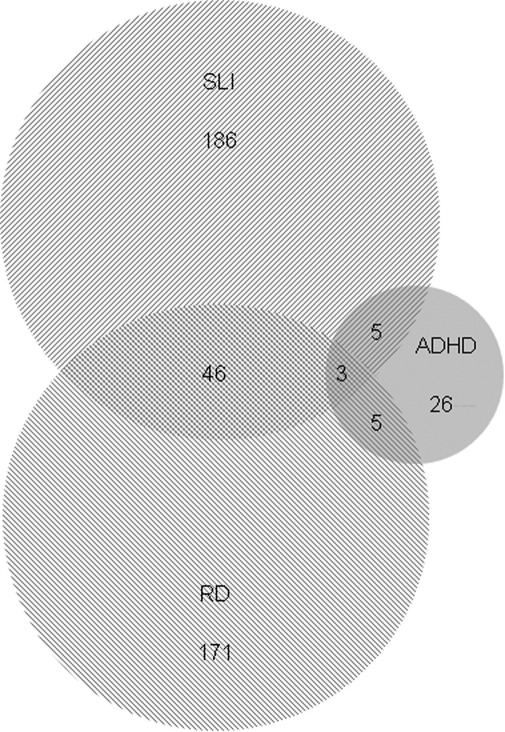
A Venn diagram illustrating the distributions of the cases identified for reading disabilities (RD), specific language impairment (SLI), and attention-deficit/hyperactivity disorder (ADHD) from Sample F1. Circle size is proportional to sample size, and circle overlaps represent comorbidity.

The ALSPAC children were assigned to one of eight affection status subgroups (Methods in Supplement 1); unaffected, RD, SLI, ADHD, or one of the four comorbid combinations (Figure 2, Table 1). From the initial sample, we filtered sequentially for missing data, ethnicity, performance IQ, and signs of autism, selecting 3725 individuals to calculate disorder prevalences. The prevalence of RD (6.04%) and SLI (6.44%) in this subgroup of the ALSPAC cohort is comparable to other studies (2,58), and the prevalence of ADHD (1.05%) is lower than previous reports of approximately 5% (59). The low prevalence of ADHD is explained primarily by a conservative assignment criterion but a specific dropout of children with ADHD from the ALSPAC study has also been suggested (60). Levels of comorbidity (Table 1) were comparable to other studies (1,9), but our conservative criterion for ADHD would have an impact on the rates of comorbidity with ADHD in this sample.

The quantitative measures selected for either ascertainment criteria or association analysis (Table 2 and Table S1 in Supplement 1) show various degrees of correlation (Table S2 in Supplement 1). A strong correlation (.514 ≤ *r* ≤ .814) was observed across the reading-related measures (excluding MEMSPAN). Low correlation was observed across the language-related measures (.099 ≤ *r* ≤ .197), and NW_REPT showed higher correlation with the reading measures. This is consistent with observations in our cohort of families with SLI (36). This also fits with the notion that the different language tests measure distinct language components and identify different groups of impaired individuals (49).

### Quantitative Genetic Analysis

We analyzed 19 SNPs for association with READ and NW_REPT to replicate previous findings with RD and SLI, respectively (Table S3 in Supplement 1). This analysis was conducted in the F1 sample (Figure 1). *DCDC2*, *KIAA0319*, and *CMIP* showed associations with READ (Table 3 and Table S4 in Supplement 1). The association with rs2143340 (*KIAA0319*) was statistically significant (*p* < .0023) and in the same direction as previously reported. The only signal observed for NW_REPT was with the *DCDC2* rs793862 marker (*p* = .03).

To follow up the results observed with READ and NW_REPT, we analyzed the SNPs showing *p* values < .05 with the other available reading and language-related measures (Table S5 in Supplement 1). We detected the strongest associations with SPELL (*DCDC2*, *KIAA0319*, and *CMIP*; Table 4) and other weak signals with NW_READ (*DCDC2* and *KIAA0319*; minimum *p* = .01) and MEMSPAN (*CMIP*; minimum *p* = .03). *DCDC2* yielded slightly stronger associations with SPELL than READ, consistent with previous findings where *DCDC2* was originally identified in a sample of individuals with spelling impairments (31). This analysis suggests that the *KIAA0319*, *DCDC2*, and *CMIP* genes contribute specifically to reading abilities and in particular to single-word reading and single-word spelling tests.

We then tested whether the associations with READ and SPELL in this population cohort were driven by the inclusion of impaired individuals (Figure 1; Methods in Supplement 1). First we tested a sample that retained the unaffected and RD cases but excluded any cases with pure SLI and/or pure ADHD (F2). Then we removed the cases of RD that had comorbidity with SLI, ADHD, or both (F3). Finally, we tested for association in the unaffected group only (F4). This analysis revealed different patterns of association underlying the results detected in F1 (Table 4 and Table S4 and S6 in Supplement 1). Specifically, the data show that the *DCDC2* associations are indeed driven by the small proportion of individuals with RD. For example, rs793862 is associated with READ (*p* = .004) and SPELL (*p* = .003) in the subgroup including all cases with RD (F2) and showing similar signal strength to F1 (*p* = .006, READ; *p* = .003, SPELL). The associations become progressively weaker when removing the approximately 50 RD cases comorbid with SLI or ADHD (*p* ∼.01; F3) and in the unaffected group (F4; minimum *p* = .03). Conversely, the SNPs that showed the strongest associations at the *KIAA0319* locus (rs2143340) and in *CMIP* (rs6564903) had similar effect sizes in the different subgroups with little variation from F1 to F4. Two other SNPs in *CMIP* (rs12927866 and rs16955705) showed the same pattern. However, two of the other four SNPs tested in *KIAA0319* (rs6935076 and rs9461045) showed modest associations with a pattern similar to *DCDC2*, where association disappeared in the unaffected subgroup (F4).

The associations with *DCDC2* show the same allelic trends as previously reported (Table 3 and Table S3 in Supplement 1). This is also the case for *KIAA0319*, with the exception of rs6935076, which showed the opposite trend from the original report in a UK sample of individuals with RD (24). This is surprising because the major allele of rs6935076, which we found to be associated with poor reading, is in high linkage disequilibrium with all the other associated alleles at this locus in populations of European descendent (ALSPAC and our cohort of dyslexic individuals). Regarding *CMIP*, the original study was conducted in two samples: a cohort of individuals with SLI and a subgroup derived from ALSPAC for being language impaired. The associations showed opposite trend between these two samples (36). Our present associations show a trend consistent with the original report in that ASLPAC subgroup.

In summary, our data suggest that *DCDC2* has a specific effect on RD, while *CMIP* and one variant at the *KIAA0319* locus (rs2143340) are significantly associated with general reading abilities. The actual location of rs2143340 is within the gene *TTRAP*, but it is in linkage disequilibrium with *KIAA0319* variants and is tagging the risk haplotype that originally refined the association to *KIAA0319* (26). The other two *KIAA0319* markers, showing an association pattern suggestive of a more specific role in RD, are instead located in the first intron (rs6935076) or regulatory sequences (rs9461045) of *KIAA0319*.

As well as positive findings, we also report lack of replications. We could not detect associations between NW_REPT and the language candidates *CMIP* and *ATP2C2* in the general population. This is consistent with our previous study, which found associations with nonword repetition for *CMIP* and *ATP2C2* only in a subgroup of individuals with language impairment (36). Our data do not support the role of the *MRPL19/C2ORF3* locus in influencing reading abilities. *MRPL19/C2ORF3* was tested using both single markers and haplotypes according to previous reports (35), but none showed any associations (haplotype analysis not shown).

### Case−Control Analysis

To test directly for association between the candidate genes and RD or SLI, we analyzed the 19 SNPs in a case−control setting. We used four subgroups of cases against a unique control group (see Methods and Materials; Figure 1). The different subgroups of cases included individuals with SLI only, RD only, SLI including cases showing comorbidity for RD and/or ADHD, and RD including cases showing comorbidity for SLI and/or ADHD. The strongest associations were observed for *DCDC2* (Table 5 and Table S7 in Supplement 1) in the cases selected for RD and including individuals with comorbidity with SLI and ADHD (minimum *p* = .003). Other association signals were observed for *KIAA0319* in the RD cases regardless of comorbidity with SLI and ADHD.

These results complement the findings observed in the quantitative analysis and support the idea that *DCDC2* is associated with RD. The SNPs rs793862 and rs807724 consistently showed the strongest associations for *DCDC2* in both the quantitative and the case−control analysis. This also agrees with our recent case−control analysis of these candidate genes in samples of individuals with RD where the *DCDC2* rs807724 marker showed the strongest association (rs793862 was not tested) (52). The case−control analysis of *KIAA0319* also agrees with the quantitative analysis, but the associations were of modest size. Associations in the case−control analysis were detected for rs6935076 and rs9461045 in the RD samples; interestingly, both these SNPs showed an association pattern in the quantitative analysis suggestive of a specific effect on RD. Conversely, the rs2143340 marker, which showed the strongest signal in the quantitative analysis and was associated with variation in the normal range, was not associated with RD in the case−control analysis.

The only other observed signal was for the *MRPL19/C2ORF3* locus showing a weak association with SLI. Our analysis found no role of *MRPL19/C2ORF3* in contributing to RD, nor was there any evidence that *CMIP* or *ATP2C2* influenced SLI.

In summary, our case−control analysis provides support for *DCDC2* and suggestive evidence for *KIAA0319* as candidate genes for RD. Inclusion of cases showing comorbidity between RD and SLI or ADHD contributed to the association signals.

## Discussion

We have described a genetic association analysis of candidate genes for RD and SLI based on the ALSPAC children cohort. The large sample size made it possible to conduct the analysis in different sample subgroups to answer specific questions. First, we sought to replicate associations reported in clinical samples, and then we tested whether these associations are detectable with specific or multiple measures to understand whether shared genetic effects contribute to the comorbidity observed between RD and SLI. Our findings support association between *DCDC2*, *KIAA0319*, and *CMIP* specifically with reading measures, but not for associations of *MRPL19/C2ORF3* with RD nor of *ATP2C2* or *CMIP* with language measures. We did not detect any pleiotropic effect, which could partly explain the comorbidity between RD and SLI, although *CMIP*, selected as a candidate for language disorder, showed association with reading.

Our strategy tested whether associations were driven by the most severe individuals. We assessed the contribution to associations of individuals that meet criteria for disorder diagnosis by removing them from the quantitative analysis (Table 4) or evaluating them directly in case−control tests (Table 5). To the best of our knowledge this is the first study that used such a strategy. The association signals we detected were supported by complementary results obtained in the two types of analysis. We show that rs2143340, the most strongly associated marker at the *KIAA0319* locus, and *CMIP* variants are significantly associated with reading and spelling skills regardless of the inclusion of the RD individuals. Consistently, these SNPs did not show associations in the case−control analysis, supporting the hypothesis that these variants contribute to reading ability variation in the normal range. Conversely, the associations detected for *DCDC2* are driven by the most impaired individuals; the associations disappear from the quantitative analysis when RD cases are removed and *DCDC2* showed the strongest associations in case−control analysis. These findings suggest that *DCDC2* is associated specifically with RD. A pattern similar to *DCDC2* is observed for two *KIAA0319* markers. Interestingly, rs9461045, one of these two markers, has a functional effect on the expression of *KIAA0319* (29). One could speculate that different genetic variants at the *KIAA0319* locus have different effects with some variants involved in the general reading processes and other directly involved in RD.

Association between reading abilities in the general population and *KIAA0319* and *DCDC2* have been reported in previous studies (42,43), including our own analysis of *KIAA0319* in ALSPAC (41). It would be interesting to see whether similar patterns will be observed in the Australian sample (42,43) when removing the most severely impaired individuals.

This is the first study reporting an effect of *CMIP* on the reading abilities of the general population. We previously analyzed *CMIP* and *ATP2C2* in the ALSPAC sample and reported an association with nonword repetition for both genes but only in a specific subgroup of language impaired individuals (36). Both quantitative and case−control analyses were carried out within that specific subgroup, the latter by comparing the two tails of the phenotypic distribution. In that study, we also failed to detect any effect of *CMIP* and *ATP2C2* on language skills in the entire ALSPAC cohort. Therefore, it is possible that the associations between nonword repetition and *CMIP* and *ATP2C2* can only be detected on a background of language impairment. This is consistent with our current findings suggesting that these two genes cannot be considered as general susceptibility factors for SLI. The association between *CMIP* and reading instead represents a direct replication of our recent findings showing that *CMIP* is associated with reading measures in the same SLI cohort where it was originally found associated with nonword repetition (52).

It has been shown that SLI and RD share a common high heritability if the child had poor nonword repetition abilities (50,61). Therefore, we might expect to see evidence of overlapping genetic associations for RD and SLI that might only become apparent in samples with specific deficits. It might be possible that the same *CMIP* variants have an effect on both reading and language problems depending on the presence of other risk factors. Our data do not support a pleiotropic effect of *KIAA0319* on reading and language-related measures as reported previously (51,52). One possible explanation is that the previously reported associations between *KIAA0319* and language skills were confined to individuals selected as language-impaired. Another explanation could be the use of psychometric tests not available in ALSPAC. These included the Omnibus language test (62) reported by Rice *et al.* (51) and measures of expressive and receptive language based on the scales of the Clinical Evaluation of Language Fundamentals (CELF-R) (63) reported by Newbury *et al.* (52).

It is striking how the associations detected in this study are specific to the single-word reading and spelling tests and not to other reading or language-related measures, despite the correlation across measures. This observation does not exclude that *KIAA0319*, *DCDC2*, and *CMIP* affect additional cognitive functions not tested here. Nevertheless this is interesting in relation to the biological function proposed for some of these genes. *KIAA0319* and *DCDC2* have been shown to play a role during the development of the cerebral cortex by regulating neuronal migration, a critical step of cortex development (64). Defects in neuronal migration lead to several human syndromes with various degrees of symptoms from epilepsy to mental retardation (65). It is therefore notable that genes involved in such a general process can lead to specific disorders rather than have a broad impact on cognition or behavior. Subtle neuronal migration defects have been suggested to be causative of RD (66). With the data reported here, we reinforce the idea that *KIAA0319* and *DCDC2*, with proven roles in neuronal migration, affect specific phenotypes.

Another important observation stems from our ability to test the effect of comorbidity on association analysis. We were able to show for the first time that inclusion of individuals with comorbid RD and SLI or ADHD do not weaken the association but rather can strengthen it, as in the case−control analysis of *DCDC2* (Table 5). This may result from an increase in sample size by including comorbid cases. Given previous reports of associations between *DCDC2* and ADHD (55), it is also possible that the associations we observe here for this gene are the combined effects of this gene on RD and ADHD separately. We could not test this hypothesis here because our ADHD sample was small (*n* = 39). In either case, these findings have an important implication. It is common practice to exclude individuals with SLI and ADHD when designing RD genetic studies to obtain samples as homogeneous as possible and to avoid confounding effects. Our data suggest that the same genes contribute to reading impairment even in the background of different disorders. This would imply also that the same cognitive deficit is at the basis of reading problems regardless of other clinical diagnoses. Providing that our observations are valid for other RD susceptibility genes, we suggest that individuals with RD comorbid for SLI or ADHD should not be excluded when designing genetic studies of RD, and their inclusion could improve sample power.

## Figures and Tables

**Table 1:** Affection Status Groups of All the Individuals from F1

Affection Status	Frequency	%
Unaffected	3283	88.13
RD	171	4.59
SLI	186	4.99
ADHD	26	.70
RD and SLI	46	1.23
RD and ADHD	5	.13
SLI and ADHD	5	.13
RD, SLI, and ADHD	3	.08

ADHD, attention-deficit hyperactivity disorder; RD, reading disability; SLI, specific language impairment.

**Table 2:** Description of Phenotypic Measures

Measure	Assignment/Phenotype^a^	Summary Description	Target Age	Reference
READ^b^	A/P	Single-word reading accuracy	7.5 year	67
READ@9	A	Single-word reading accuracy	9.5 year	68
SPELL	P	Single-word spelling accuracy	7.5 year	68
PHONEME	P	Phoneme awareness	7.5 year	69
NW-READ	P	Single-non-word reading accuracy	9.5 year	68
MEMSPAN	P	Working memory	10.5 year	70
WOLD	A/P	Listening and comprehension test	8.5 year	71
NW-REPT^c^	A/P	Phonological short-term memory test	8.5 year	72
CCC-SUM7	A/P	Sum of first seven scales from Children's Communication Checklist	7.5 year	73
Speech/language therapy	A	Child has ever had speech/language therapy	7.6 year	
DAWBA DSM-IV	A	Attention-deficit hyperactivity disorder diagnosis	7.6 year−8.5 year	74
PERF_IQ	A	Performance IQ	8.5 year	75

See Table S1 in Supplement 1 for more details.

a Specifies whether the measure was used for assignment (A) of case status or as phenotype (P) for quantitative analysis.

b Core measure for RD.

c Core measure for specific language impairment.

**Table 3:** Associations Results of the 19 SNPs Tested in F1 with READ and NW_REPT

			READ				NW-REPT				
Chr.	Gene Locus	SNP	*n*	β	SE	*p*	*n*	β	SE	*p*	Risk Allele
2	*MRPL19/C2ORF3*	rs1000585	3,050	.00	.03	.972	3,048	.00	.03	.928	
2	*MRPL19/C2ORF3*	rs917235	3,165	.00	.03	.949	3,163	−.02	.03	.353	
2	*MRPL19/C2ORF3*	rs714939	3,041	.02	.03	.427	3,039	.01	.03	.646	
6	*DCDC2*	rs793862	3,117	−.08	.03	.006	3,115	−.06	.03	.031	A (minor)
6	*DCDC2*	rs807701	3,193	−.05	.03	.033	3,191	−.03	.03	.185	G (minor)
6	*DCDC2*	rs807724	3,085	−.07	.03	.015	3,083	−.03	.03	.257	C (minor)
6	*DCDC2*	rs1087266	3,198	−.03	.03	.219	3,196	.00	.03	.915	
6	*KIAA0319*	rs761100	3,190	−.03	.03	.211	3,188	−.01	.03	.603	
6	*KIAA0319*	rs6935076	3,006	.07	.03	.011	3,004	.02	.03	.482	G (major)^a^
6	*KIAA0319*	rs2038137	3,053	−.02	.03	.374	3,051	−.02	.03	.544	
6	*KIAA0319*	rs9461045	3,126	−.08	.03	.024	3,124	−.03	.03	.368	T (minor)
6	*KIAA0319*^b^	rs2143340	3,042	−.11	.04	**.001**	3,040	−.04	.04	.242	G (minor)
16	*CMIP*	rs12927866	3,055	−.07	.03	.005	3,053	−.04	.03	.136	T (minor)^a^
16	*CMIP*	rs6564903	3,157	−.08	.02	.002	3,155	−.02	.02	.360	T (minor)^a^
16	*CMIP*	rs4265801	3,052	.02	.03	.449	3,050	.03	.03	.289	
16	*CMIP*	rs16955705	3,050	−.06	.03	.029	3,048	−.02	.03	.482	C (minor)^a^
16	*ATP2C2*	rs16973771	3,009	.01	.03	.691	3,007	.02	.03	.493	
16	*ATP2C2*	rs2875891	3,049	.00	.03	.950	3,047	.02	.03	.458	
16	*ATP2C2*	rs8045507	3,046	.00	.03	.979	3,044	.01	.03	.588	

Only one *p* value was statistically significant (< .0023; Methods in Supplement 1) and is highlighted in bold; β (beta) values are standardized and relative to the minor allele (as defined in Table S3 in Supplement 1). Risk allele is reported only for markers showing *p* values < .05. SNP, single nucleotide polymorphism.

a Opposite trend compared with original reports (24,36).

b Within *TTRAP*.

**Table 4:** Summary of Results Showing Association (*p* < .05) with Quantitative Measures

Chr.	Gene Locus	SNP	F1				F2				F3				F4: Unaffected				Risk Allele
			READ																
			N	β	SE	P	N	β	SE	P	N	β	SE	P	N	β	SE	P	
6	*DCDC2*	rs793862	3,117	–.08	.03	.006	2,936	–.09	.03	.004	2,890	–.08	.03	.010	2,740	–.06	.03	.042	A (minor)
6	*DCDC2*	rs807701	3,193	–.05	.03	.033	3,003	–.04	.03	.090	2,954	–.03	.03	.276	2,803	–.02	.02	.376	G (minor)
6	*DCDC2*	rs807724	3,085	–.07	.03	.015	2,898	–.07	.03	.018	2,850	–.05	.03	.091	2,700	–.02	.03	.422	C (minor)
6	*KIAA0319*	rs6935076	3,006	.07	.03	.011	2,831	.08	.03	.003	2,784	.07	.03	.006	2,646	.05	.02	.028	G (major)^a^
6	*KIAA0319*	rs9461045	3,126	–.08	.03	.024	2,947	–.08	.03	.026	2,901	–.08	.03	.022	2,752	–.05	.03	.162	T (minor)
6	*KIAA0319*^b^	rs2143340	3,042	–.11	.04	**.001**	2,864	–.12	.04	**.001**	2,817	–.12	.04	**.001**	2,677	–.11	.03	**.001**	G (minor)
16	*CMIP*	rs12927866	3,055	–.07	.03	.005	2,874	–.08	.03	.004	2,829	–.07	.03	.005	2,690	–.07	.02	.005	T (minor)^a^
16	*CMIP*	rs6564903	3,157	–.08	.02	.002	2,966	–.08	.03	**.002**	2,919	–.08	.03	**.002**	2,768	–.07	.02	.002	T (minor)^a^
16	*CMIP*	rs16955705	3,050	–.06	.03	.029	2,869	–.06	.03	.022	2,824	–.06	.03	.019	2,684	–.05	.02	.027	C (minor)^a^
			SPELL																
6	*DCDC2*	rs793862	3,094	–.09	.03	.003	2,913	–.09	.03	.003	2,871	–.08	.03	.009	2,729	–.06	.03	.030	A (minor)
6	*DCDC2*	rs807724	3,065	–.08	.03	.007	2,878	–.08	.03	.011	2,834	–.06	.03	.050	2,691	–.04	.03	.204	C (minor)
6	*KIAA0319*^b^	rs2143340	3,023	–.10	.04	.004	2,845	–.10	.04	.005	2,802	–.11	.04	.004	2,669	–.10	.04	.006	G (minor)
16	*CMIP*	rs12927866	3,036	–.06	.03	.014	2,855	–.07	.03	.009	2,814	–.07	.03	.011	2,682	–.06	.03	.014	T (minor)^a^
16	*CMIP*	rs6564903	3,136	–.07	.02	.008	2,945	–.07	.03	.003	2,901	–.07	.03	.004	2,758	–.07	.02	.008	T (minor)^a^
16	*CMIP*	rs16955705	3,030	–.06	.03	.026	2,849	–.06	.03	.019	2,808	–.06	.03	.017	2,675	–.06	.03	.027	C (minor)^a^

Only SNPs showing *p* values < .05 in any group tested are reported; *p* values statistically significant (< .0023; Methods in Supplement 1) are in bold; β (beta) values are standardized and relative to the minor allele (as defined in Table S3 in Supplement 1). SNP, single nucleotide polymorphism.

a Opposite trend compared with original reports (24,36).

b Within *TTRAP.*

**Table 5:** Summary of the Results of the Case−Control Analysis

Chr.	Gene Locus	SNP	No. of Controls	SLI Only			SLI and Comorbid Cases			RD Only			RD and Comorbid Cases			
				*n*	*p*	Odds Ratio	*n*	*p*	Odds Ratio	*n*	*p*	Odds Ratio	*n*	*p*	Odds Ratio	Risk Allele
2	*MRPL19/C2ORF3*	rs917235	375	162	.033	1.33	211	.103	1.22	148	.610	1.07	197	.672	1.06	G (minor)
6	*DCDC2*	rs793862	375	155	.418	1.13	201	.101	1.26	150	.021	1.42	196	.005	1.47	a (minor)
6	*DCDC2*	rs807701	379	161	.173	1.21	210	.016	1.36	151	.173	1.21	200	.018	1.36	G (minor)
6	*DCDC2*	rs807724	371	158	.754	1.05	206	.146	1.24	150	.035	1.40	198	.003	1.52	C (minor)
6	*KIAA0319*	rs6935076	363	149	.993	1.00	196	.661	.95	138	.026	.72	185	.011	.71	G (major)^a^
6	*KIAA0319*	rs9461045	375	153	.692	1.08	199	.561	1.10	149	.026	1.47	195	.035	1.40	T (minor)

RD, reading disability; SLI, specific language impairment; SNP, single nucleotide polymorphism. Only SNPs showing *p* values < .05 in any of group tested are reported.

a Opposite trend compared with original report (24).
