# Supplementary material for: DCDC2, KIAA0319 and CMIP Are Associated with Reading-Related Traits
Source: Biol Psychiatry. 2011 Aug 1;70(3):237–45. doi: 10.1016/j.biopsych.2011.02.005 (PMC3139836; doi:10.1016/j.biopsych.2011.02.005)
Supplement: Supplement 1 [file mmc1.pdf]

Supplemental Information

Methods and Materials

Sample description

The ALSPAC cohort consists of over 15,000 children from the southwest of England that had expected dates of delivery between 1st April 1991 and 31st December 1992 (1). From age 7, all children were invited annually for assessments on a wide range of physical, behavioral, and neuropsychological traits, including reading and language-related measures. DNA is available for approximately 11,000 ALSPAC children. Informed written consent was obtained from the parents after receiving a complete description of the study at the time of enrollment into the ALSPAC project, with the option for them or their children to withdraw at any time. Ethical approval for the present study was obtained from the ALSPAC Law and Ethics Committee and the Local Research Ethics Committees.

Phenotypes and sample subgroups

We selected a range of quantitative measures which are comparable to the psychometric tests commonly used to ascertain probands and conduct quantitative analysis in genetic studies of RD and SLI (Table 1 and S1).

From the entire ALSPAC children cohort (N = 15,211) we identified a sample that included only individuals with a near complete data set on all the measures used for sample assignment, IQ and ethnicity (N = 4,761). To avoid effects of population stratification, we excluded individuals that did not have a white European ethnicity based on four different assessments. Then we excluded individuals with a low performance IQ (PERF_IQ < 85) or a score < -3 SD for CCC_SUM7; this second filter was to rule out individuals with autistic features. These exclusion criteria removed individuals that may have performed badly on the psychometric tests for reasons other than specific reading or language impairment. This strategy left us a sample (F1; N = 3,725) on which we based our initial analysis (Figure 1). Individuals were then assigned to the groups of RD, SLI, ADHD, any of the four comorbid combinations of these three disorders, or else unaffected. RD was identified if the child scored < -1 SD on tests of single-word reading at 7 and 9 years, which is the most commonly used measure to ascertain individuals with RD. Two time points were used to correct for random error on each individual

measure. To capture the different components of language impairment, an assignment of SLI was given if an individual scored positive for at least two of the following four criteria: i) CCC_SUM7 < -1 SD, ii) NW_REPT < -1 SD, iii) WOLD < -1 SD, and iv) positive response on speech/language therapy questionnaire. These four criteria target different aspects of language problems (Table S1) and while each of them might over-identify impairment, two concomitant low scores have been shown to be a valid strategy to predict clinical diagnosis (2). An assignment of ADHD was based on a DAWBA DSM-IV clinical diagnosis. Comorbidity was assigned if any child met the criteria for more than one affection status. In total, there were 442 affected individuals who met any of the assignment criteria detailed above, of whom 276 were male and 166 were female (ratio of 1.663 males to 1 female). There were also 3,283 unaffected individuals, of which 1,523 were male and 1,760 were female (ratio of 0.865 males to 1 female). From the 3,283 unaffected individuals we selected a group of controls to carry out case-control analysis. We chose individuals that had a score greater than the mean for all the quantitative measures used to assign an affection status. A total of 595 individuals fulfilled these criteria (276 males and 319 females). From these, we randomly selected 166 females from the 319 available to produce a final control group of 442 unaffected individuals with a sex ratio matching that of the overall affected individuals.

From F1 we identified two different subgroups to specifically test the effect of comorbidity. In the first subgroup (F2; N = 3,508) we excluded pure SLI (N = 186), pure ADHD (N = 26) cases and comordid cases of SLI and ADHD (N = 5) thereby retaining all cases of RD and the unaffected individuals. Individuals comorbid for RD and SLI (N = 46), RD and ADHD (N = 5) or RD, SLI and ADHD (N = 3) were then removed so that the final sample comprised the unaffected individuals and cases with pure RD (F3; N = 3454). The exclusion of pure RD cases from F3 resulted in the 3,283 unaffected individuals (F4).

Multiple test correction

We analyzed the 19 SNPs that passed quality control criteria (Table S3) for two quantitative measures selected to match previous findings in the literature. These SNPs lie within 11 clusters of inter-marker correlations (LD blocks) which had at least 8 pairs of SNPs in strong LD (r^2 > 0.6). The LD blocks were independently established with both PLINK on our data from sample F1, and HaploView version 4.2 (3) on CEPH HapMap data. Therefore, we applied a

multiple test correction for 22 independent tests (11 SNP clusters and 2 phenotypes) to a significance level of $P = 0.05$ resulting in $P = 0.0023$. It should also be noted that this ALSPAC cohort has been tested previously for other SNPs and phenotypes, therefore we should consider these additional tests in calculating a significant threshold *p*-value, or else use the genome-significant threshold of 5×10^{-8}. However, this is far too conservative and the goal of this study is to investigate the effect of established associations on specific phenotypic components rather than conducting a discovery exercise. Therefore, while it is important to interpret our data in the light of multiple testing, we show all the association results to allow evaluation of any patterns of association and we define as “statistically significant” only *p*-values which meet the corrected level of association at $P < 0.0023$.

Table S1. Descriptive details of phenotypic measures

Measure	Assignment/ Phenotype	Summary Description	Target Age	Details	Source
READ*	A/P	Single-word reading accuracy	7.5 yr	The child was asked to read aloud a series of 48 unconnected words which increased in difficulty.	Rust J, Golombok S, Trickey G (1993): *WORD: Wechsler Objective Reading Dimensional Manual.* Sidcup, UK: Psychological Corporation
READ@9	A	Single-word reading accuracy	9.5 yr	This was assessed by asking the child to read out loud ten real words, followed by ten non-words.	Nunes T, Bryant P, Olsson J (2003): Learning morphological and phonological spelling rules: An intervention study. *Sci Stud Read.* 7:298-307
SPELL	P	Single-word spelling accuracy	7.5 yr	The child was asked to spell a series of 15 regular and irregular words of increasing difficulties.	Nunes T, Bryant P, Olsson J (2003): Learning morphological and phonological spelling rules: An intervention study. *Sci Stud Read*. 7:298-307
PHONEME	P	Phoneme awareness	7.5 yr	The phoneme deletion task (Auditory Analysis Test) comprised 2 practice and 40 test items of increasing difficulty. The task involved asking the child to repeat a word and then to say it again but with part of the word (a phoneme or number of phonemes) removed.	Rosner J, Simon, DP (1971): The auditory analysis test: an initial report. *J Learn Disabil*. 4:40-48
MEMSPAN	P	Working memory	10.5 yr	Working memory was tested using the Counting Span Task, which requires the simultaneous processing and storage of information. On the computer monitor the child was presented with a number of red and blue dots on a white screen. The child was asked to point to and count the number of red dots out loud (the processing component). After each set, the child was asked to recall the number of red dots seen on each screen in the order they were presented within that set (the storage component).	Case R, Kurland DM, Goldberg J (1982): Operational efficiency and the growth of short-term memory span. *J Exp Child Psychol*. 33
WOLD	A/P	Listening and comprehension test	8.5 yr	The child was read a paragraph about a picture, which the child is shown. The child then answers questions on what he/she has heard. The child has to make inferences about what was read to him/her and answer the questions verbally. The task was discontinued if the child got three consecutive questions incorrect.	Rust J (1996): *WOLD Wechsler Objective Language Dimensions Manual.* London, UK: The Psychological Corporation
NW_REPT**	A/P	Phonological short term memory test	8.5 yr	An adaptation of the Nonword Repetition Test was used. This comprised twelve nonsense words, four each of 3, 4 and 5 syllables and conforming to English rules for sound combinations. The child was asked to listen to each word via an audio cassette recorder and then repeat each item.	Gathercole SE, Willis CS, Baddeley AD, Emslie H (1994): The Children's Test of Nonword Repetition: a test of phonological working memory. *Memory*. 2:103-127

Measure	Assignment/ Phenotype	Summary Description	Target Age	Details	Source
CCC_SUM7	A/P	Sum of 1st 7 scales from Children's Communication Checklist	7.5 yr	The CCC consists of 70 items grouped into 9 subscales with scores defined for each subscale as well as a summary score for pragmatic aspects of communication as the sum of the 3rd to 7th subscales. In this questionnaire the first 53 items making up the first 7 subscales were used.	Bishop DV (1998): Development of the Children's Communication Checklist (CCC): a method for assessing qualitative aspects of communicative impairment in children. *J Child Psychol Psychiatry*. 39:879-891
Speech/Language Therapy	A	Child has ever had speech/ language therapy	7.6 yr	This questionnaire was sent out to mothers when their study child was 91 months old.	N/A
DAWBA DSM-IV	A	DAWBA DSM-IV - any ADHD	7.6 yr - 8.5yr	Diagnosis of ADHD was based on the answers to a set of questionnaires given to the parents (at 91 months) and a teacher report if available (at YEAR 3). Full DSM-IV diagnoses were only made for children for whom the parent report was available.	Goodman R, Ford T, Richards H, Gatward R, Meltzer H (2000): The Development and Well-Being Assessment: description and initial validation of an integrated assessment of child and adolescent psychopathology. *J Child Psychol Psychiatry*. 41:645-655
PERF_IQ	A	Performance IQ	8.5 yr	The WISC-III UK was used to assess cognitive function. A short form of the measure was employed where alternate items (always starting with item number 1 in the standard form) were used for all subtests, with the exception of the coding subtest which was administered in its full form.	Wechsler D, Golombok S, Rust J (1992): *WISC-IIIUK: Wechsler Intelligence Scale for Children.* Sidcup, UK: The Psychological Corporation
NW_READ	P	Single-non-word reading accuracy	9.5 yr	This was assessed by asking the child to read out loud ten real words, followed by ten non-words.	Nunes T, Bryant P, Olsson J (2003): Learning morphological and phonological spelling rules: An intervention study. *Sci Stud Read*. 7:298-307

* core measure for dyslexia
** core measure for SLI

Table S2. Correlation of the quantitative measures used in this study based on sample F1

	WOLD	NW_REPT	CCC_SUM7	READ	READ@9	SPELL	PHONEME	NW_READ	MEMSPAN
**NW_REPT**	0.197								
**CCC_SUM7**	0.099	0.183							
**READ**	0.253	0.399	0.224						
**READ@9**	0.195	0.348	0.201	0.711					
**SPELL**	0.189	0.332	0.203	0.814	0.644				
**PHONEME**	0.163	0.360	0.187	0.669	0.536	0.644			
**NW_READ**	0.151	0.304	0.154	0.646	0.695	0.616	0.514		
**MEMSPAN**	0.090	0.208	0.114	0.275	0.241	0.285	0.268	0.235	
**PERF_IQ**	0.184	0.160	0.096	0.243	0.172	0.201	0.194	0.161	0.181

Dotted lines separate the reading, the language and the IQ measures.

All correlations significant at 0.01 level (1-tailed test).

Table S3. SNPs passing QC criteria

Chr.	Gene locus	Genetic feature	SNP name	LD block[a]	Base pair position	After removing samples with >25% missing genotype data					Risk allele from previous studies	References of original associations
						F0	F1	F1	F1	F1		
						Genotyping success[b]	Minor[c]	Major[c]	MAF[c]	HWE P[c]		
2p12	*MRPL19/C2ORF3*	intergenic	rs1000585	1	75,676,670	0.938	G	A	0.399	0.443	G	(4)
2p12	*MRPL19/C2ORF3*	intergenic	rs917235	2	75,679,327	0.980	G	A	0.465	0.307	G	(4)
2p12	*MRPL19/C2ORF3*	intergenic	rs714939	3	75,688,615	0.933	A	G	0.381	0.491	G	(4)
6p22.3	*DCDC2*	intronic	rs793862	4	24,315,179	0.927	A	G	0.258	0.589	A	(5)
6p22.3	*DCDC2*	intronic	rs807701	5	24,381,770	0.984	G	A	0.340	0.054	G	(6)
6p22.3	*DCDC2*	intronic	rs807724	5	24,386,848	0.961	C	T	0.213	0.098	NR	(5)
6p22.3	*DCDC2*	intronic	rs1087266	6	24,463,129	0.985	A	G	0.446	0.798	NR	(5)
6p22.3	*KIAA0319*	intronic	rs761100	7	24,740,621	0.982	A	C	0.438	0.086	C	(7)
6p22.3	*KIAA0319*	intronic	rs6935076	7	24,752,301	0.924	A	G	0.373	0.402	A	(8)
6p22.3	*KIAA0319*	intronic	rs2038137	7	24,753,922	0.940	T	G	0.373	0.460	G	(9)
6p22.3	*KIAA0319*	intergenic	rs9461045	7	24,757,040	0.926	T	C	0.173	1.000	T	(10)
6p22.3	*KIAA0319*[d]	intronic	rs2143340	8	24,767,050	0.938	G	A	0.151	0.613	G	(9)
16q23.2	*CMIP*	intronic	rs12927866	9	80,209,823	0.939	T	C	0.406	0.303	C	(11)
16q23.2	*CMIP*	intronic	rs6564903	9	80,211,158	0.973	T	C	0.469	0.337	C	(11)
16q23.2	*CMIP*	intronic	rs4265801	10	80,222,553	0.938	T	G	0.455	0.257	T	(11)
16q23.2	*CMIP*	intronic	rs16955705	10	80,230,851	0.939	C	A	0.465	0.409	A / C[e]	(11)
16q24.1	*ATP2C2*	intronic	rs16973771	11	83,018,079	0.928	C	T	0.408	0.115	T	(11)
16q24.1	*ATP2C2*	intronic	rs2875891	11	83,021,410	0.939	T	C	0.363	0.852	C	(11)
16q24.1	*ATP2C2*	intronic	rs8045507	11	83,022,078	0.937	A	G	0.404	0.111	G	(11)

[a] blocks as defined independently by both the present ALSPAC data and the CEPH HapMap data

[b] calculated from all available individuals (F0)

[c] based on individuals after filtering for ethnicity (F1)

[d] within *TTRAP*

[e] allele "A" in families from SLIC and allele "C" in ALSPAC subgroup

HWE, Hardy-Weinberg equilibrium; LD, linkage disequilibrium; NR, not reported; MAF, minor allele frequency; QC, quality control; SNP, single nucleotide polymorphism.

Table S4. Results of association analysis of initial SNPs panel with READ

Chr.	Gene locus	Genetic Feature	SNP name	F1				F2				F3				
				Number	β	SE	P	Number	β	SE	P	Number	β	SE	P	Number
2	*MRPL19/C2ORF3*	intergenic	rs1000585	3,050	0.00	0.03	0.972	2,871	0.00	0.03	0.902	2,826	0.01	0.03	0.823	2,684
2	*MRPL19/C2ORF3*	intergenic	rs917235	3,165	0.00	0.03	0.949	2,975	0.01	0.03	0.774	2,926	0.01	0.03	0.842	2,778
2	*MRPL19/C2ORF3*	intergenic	rs714939	3,041	0.02	0.03	0.427	2,860	0.01	0.03	0.588	2,816	-0.01	0.03	0.809	2,674
6	*DCDC2*	intronic	rs793862	3,117	-0.08	0.03	0.006	2,936	-0.09	0.03	0.004	2,890	-0.08	0.03	0.010	2,740
6	*DCDC2*	intronic	rs807701	3,193	-0.05	0.03	0.033	3,003	-0.04	0.03	0.090	2,954	-0.03	0.03	0.276	2,803
6	*DCDC2*	intronic	rs807724	3,085	-0.07	0.03	0.015	2,898	-0.07	0.03	0.018	2,850	-0.05	0.03	0.091	2,700
6	*DCDC2*	intronic	rs1087266	3,198	-0.03	0.03	0.219	3,009	-0.04	0.03	0.149	2,961	-0.03	0.03	0.200	2,808
6	*KIAA0319*	intronic	rs761100	3,190	-0.03	0.03	0.211	3,001	-0.04	0.03	0.117	2,953	-0.03	0.03	0.262	2,801
6	*KIAA0319*	intronic	rs6935076	3,006	0.07	0.03	0.011	2,831	0.08	0.03	0.003	2,784	0.07	0.03	0.006	2,646
6	*KIAA0319*	intronic	rs2038137	3,053	-0.02	0.03	0.374	2,874	-0.03	0.03	0.274	2,827	-0.01	0.03	0.586	2,688
6	*KIAA0319*	intergenic	rs9461045	3,126	-0.08	0.03	0.024	2,947	-0.08	0.03	0.026	2,901	-0.08	0.03	0.022	2,752
6	*KIAA0319* [a]	intronic	rs2143340	3,042	-0.11	0.04	**0.001**	2,864	-0.12	0.04	**0.001**	2,817	-0.12	0.04	**0.001**	2,677
16	*CMIP*	intronic	rs12927866	3,055	-0.07	0.03	0.005	2,874	-0.08	0.03	0.004	2,829	-0.07	0.03	0.005	2,690
16	*CMIP*	intronic	rs6564903	3,157	-0.08	0.02	0.002	2,966	-0.08	0.03	**0.002**	2,919	-0.08	0.03	**0.002**	2,768
16	*CMIP*	intronic	rs4265801	3,052	0.02	0.03	0.449	2,872	0.02	0.03	0.400	2,827	0.02	0.03	0.360	2,686
16	*CMIP*	intronic	rs16955705	3,050	-0.06	0.03	0.029	2,869	-0.06	0.03	0.022	2,824	-0.06	0.03	0.019	2,684
16	*ATP2C2*	intronic	rs16973771	3,009	0.01	0.03	0.691	2,830	0.00	0.03	0.868	2,786	0.00	0.03	0.905	2,648
16	*ATP2C2*	intronic	rs2875891	3,049	0.00	0.03	0.950	2,869	-0.01	0.03	0.746	2,824	-0.01	0.03	0.720	2,682
16	*ATP2C2*	intronic	rs8045507	3,046	0.00	0.03	0.979	2,866	-0.01	0.03	0.830	2,821	-0.01	0.03	0.838	2,680

P-values statistically significant (< 0.0023) are in bold

[a] within *TTRAP*

β (beta) values are standardized and relative to the minor allele (as defined in Table S3)

Table S5. Results of follow-up analysis of the nine SNPs showing initial associations with either READ or NW_REPT

Chr.	Gene locus	Genetic Feature	SNP name	F1: READ				F1: SPELL				F1: NW_READ				F1: PHONEME				F1: MEMSPAN				F1: NW_REPT				F1: WOLD				F1: CCC_SUM7			
				Number	β	SE	P	Number	β	SE	P	Number	β	SE	P	Number	β	SE	P	Number	β	SE	P	Number	β	SE	P	Number	β	SE	P	Number	β	SE	P
6	*DCDC2*	intronic	rs793862	3,117	-0.08	0.03	0.006	3,094	-0.09	0.03	0.003	3,116	-0.07	0.03	0.018	3,115	-0.04	0.03	0.159	2,803	-0.03	0.03	0.334	3,115	-0.06	0.03	0.031	3,117	-0.04	0.03	0.163	3,117	-0.01	0.03	0.616
6	*DCDC2*	intronic	rs807701	3,193	-0.05	0.03	0.033	3,170	-0.05	0.03	0.052	3,192	-0.04	0.03	0.163	3,191	-0.04	0.03	0.088	2,867	-0.02	0.03	0.375	3,191	-0.03	0.03	0.185	3,192	-0.04	0.03	0.126	3,193	-0.03	0.03	0.272
6	*DCDC2*	intronic	rs807724	3,085	-0.07	0.03	0.015	3,065	-0.08	0.03	0.007	3,084	-0.07	0.03	0.019	3,083	-0.05	0.03	0.129	2,762	-0.01	0.03	0.800	3,083	-0.03	0.03	0.257	3,085	-0.04	0.03	0.186	3,085	-0.02	0.03	0.509
6	*KIAA0319*	intronic	rs6935076	3,006	0.07	0.03	0.011	2,987	0.05	0.03	0.053	3,005	0.05	0.03	0.039	3,005	0.04	0.03	0.120	2,700	-0.01	0.03	0.849	3,004	0.02	0.03	0.482	3,005	0.04	0.03	0.098	3,006	-0.05	0.03	0.057
6	*KIAA0319*	intergenic	rs9461045	3,126	-0.08	0.03	0.024	3,103	-0.06	0.03	0.073	3,125	-0.06	0.03	0.058	3,124	-0.06	0.03	0.103	2,810	0.01	0.04	0.814	3,124	-0.03	0.03	0.368	3,126	0.00	0.03	0.938	3,126	0.02	0.03	0.605
6	*KIAA0319* [a]	intronic	rs2143340	3,042	-0.11	0.04	**0.001**	3,023	-0.10	0.04	0.004	3,041	-0.09	0.04	0.014	3,041	-0.05	0.04	0.154	2,733	0.01	0.04	0.778	3,040	-0.04	0.04	0.242	3,041	-0.02	0.04	0.619	3,042	0.00	0.04	0.913
16	*CMIP*	intronic	rs12927866	3,055	-0.07	0.03	0.005	3,036	-0.06	0.03	0.014	3,054	-0.05	0.03	0.052	3,054	-0.02	0.03	0.359	2,743	-0.04	0.03	0.110	3,053	-0.04	0.03	0.136	3,054	0.01	0.03	0.615	3,055	0.02	0.03	0.368
16	*CMIP*	intronic	rs6564903	3,157	-0.08	0.02	0.002	3,136	-0.07	0.02	0.008	3,156	-0.04	0.02	0.120	3,155	-0.04	0.02	0.133	2,829	-0.05	0.03	0.060	3,155	-0.02	0.02	0.360	3,156	0.00	0.02	0.993	3,157	0.01	0.02	0.793
16	*CMIP*	intronic	rs16955705	3,050	-0.06	0.03	0.029	3,030	-0.06	0.03	0.026	3,049	-0.03	0.03	0.195	3,049	-0.02	0.03	0.502	2,739	-0.06	0.03	0.032	3,048	-0.02	0.03	0.482	3,049	0.02	0.03	0.354	3,050	0.02	0.03	0.500

P-values statistically significant (< 0.0023) are in bold

[a] within *TTRAP*

β (beta) values are standardized and relative to the minor allele (as defined in Table S3)

Table S6. Complete analysis with SPELL to follow-up the nine SNPs that gave initial association with either READ or NW_REPT

Chr.	Gene locus	Genetic Feature	SNP name	F1				F2				F3				F4			
				Number	β	SE	P	Number	β	SE	P	Number	β	SE	P	Number	β	SE	P
6	*DCDC2*	intronic	rs793862	3,094	-0.09	0.03	0.003	2,913	-0.09	0.03	0.003	2,871	-0.08	0.03	0.009	2,729	-0.06	0.03	0.030
6	*DCDC2*	intronic	rs807701	3,170	-0.05	0.03	0.052	2,980	-0.04	0.03	0.117	2,935	-0.03	0.03	0.309	2,792	-0.02	0.03	0.459
6	*DCDC2*	intronic	rs807724	3,065	-0.08	0.03	0.007	2,878	-0.08	0.03	0.011	2,834	-0.06	0.03	0.050	2,691	-0.04	0.03	0.204
6	*KIAA0319*	intronic	rs6935076	2,987	0.05	0.03	0.053	2,812	0.06	0.03	0.022	2,769	0.06	0.03	0.023	2,638	0.05	0.03	0.084
6	*KIAA0319*	intergenic	rs9461045	3,103	-0.06	0.03	0.073	2,924	-0.06	0.03	0.082	2,882	-0.06	0.03	0.085	2,741	-0.03	0.03	0.332
6	*KIAA0319* [a]	intronic	rs2143340	3,023	-0.10	0.04	0.004	2,845	-0.10	0.04	0.005	2,802	-0.11	0.04	0.004	2,669	-0.10	0.04	0.006
16	*CMIP*	intronic	rs12927866	3,036	-0.06	0.03	0.014	2,855	-0.07	0.03	0.009	2,814	-0.07	0.03	0.011	2,682	-0.06	0.03	0.014
16	*CMIP*	intronic	rs6564903	3,136	-0.07	0.02	0.008	2,945	-0.07	0.03	0.003	2,901	-0.07	0.03	0.004	2,758	-0.07	0.02	0.008
16	*CMIP*	intronic	rs16955705	3,030	-0.06	0.03	0.026	2,849	-0.06	0.03	0.019	2,808	-0.06	0.03	0.017	2,675	-0.06	0.03	0.027

[a] within *TTRAP*
β (beta) values are standardized and relative to the minor alleles (as defined in Table S3)

Table S7. Complete analysis with either SLI or RD cases compared to a standard set of controls

Chr.	Gene locus	Genetic Feature	SNP name	Number of controls	Pure SLI				Pure RD				All SLI				All RD			
					Number of cases	Odds ratio	P	Risk allele	Number of cases	Odds ratio	P	Risk allele	Number of cases	Odds ratio	P	Risk allele	Number of cases	Odds ratio	P	Risk allele
2	*MRPL19/C2ORF3*	intergenic	rs1000585	361	152	1.28	0.078		142	0.99	0.951		197	1.24	0.095		187	1.03	0.821	
2	*MRPL19/C2ORF3*	intergenic	rs917235	375	162	1.33	0.033	G (minor)	148	1.07	0.610		211	1.22	0.103		197	1.06	0.672	
2	*MRPL19/C2ORF3*	intergenic	rs714939	360	154	0.94	0.668		142	0.94	0.656		198	0.81	0.099		186	0.81	0.125	
6	*DCDC2*	intronic	rs793862	375	155	1.13	0.418		150	1.42	0.021	A (minor)	201	1.26	0.101		196	1.47	0.005	A (minor)
6	*DCDC2*	intronic	rs807701	379	161	1.21	0.173		151	1.21	0.173		210	1.36	0.016	G (minor)	200	1.36	0.018	G (minor)
6	*DCDC2*	intronic	rs807724	371	158	1.05	0.754		150	1.40	0.035	C (minor)	206	1.24	0.146		198	1.52	0.003	C (minor)
6	*DCDC2*	intronic	rs1087266	378	160	0.82	0.139		153	0.89	0.414		208	0.91	0.454		201	0.96	0.733	
6	*KIAA0319*	intronic	rs761100	378	161	1.08	0.582		152	1.10	0.472		209	1.11	0.390		200	1.17	0.210	
6	*KIAA0319*	intronic	rs6935076	363	149	1.00	0.993		138	0.72	0.026	G (major)	196	0.95	0.661		185	0.71	0.011	G (major)
6	*KIAA0319*	intronic	rs2038137	366	151	1.15	0.330		139	1.08	0.599		198	1.21	0.149		186	1.18	0.204	
6	*KIAA0319*	intronic	rs9461045	375	153	1.08	0.692		149	1.47	0.026	T (minor)	199	1.10	0.561		195	1.40	0.035	T (minor)
6	*KIAA0319* [a]	intronic	rs2143340	367	150	0.90	0.603		140	1.24	0.249		197	0.95	0.778		187	1.21	0.269	
16	*CMIP*	intronic	rs12927866	360	154	0.83	0.192		139	1.06	0.663		199	0.91	0.433		184	1.07	0.601	
16	*CMIP*	intronic	rs6564903	369	162	0.89	0.392		151	1.16	0.278		209	0.96	0.714		198	1.15	0.265	
16	*CMIP*	intronic	rs4265801	360	153	1.21	0.174		141	1.07	0.656		198	1.17	0.205		186	1.08	0.560	
16	*CMIP*	intronic	rs16955705	361	154	0.82	0.140		140	1.05	0.736		199	0.86	0.237		185	1.02	0.861	
16	*ATP2C2*	intronic	rs16973771	354	152	0.89	0.396		138	0.97	0.813		196	0.90	0.434		182	0.97	0.790	
16	*ATP2C2*	intronic	rs2875891	357	153	0.84	0.229		142	0.94	0.674		198	0.87	0.286		187	0.95	0.684	
16	*ATP2C2*	intronic	rs8045507	361	153	0.88	0.347		141	0.97	0.851		198	0.91	0.446		186	0.98	0.899	

[a] within *TTRAP*

β (beta) values are standardized and relative to the minor allele (as defined in Table S3)

References

1. Golding J, Pembrey M, Jones R (2001): ALSPAC--the Avon Longitudinal Study of Parents and Children. I. Study methodology. *Paediatr Perinat Epidemiol*. 15:74-87.
2. Barry JG, Yasin I, Bishop DV (2007): Heritable risk factors associated with language impairments. *Genes Brain Behav*. 6:66-76.
3. Barrett JC, Fry B, Maller J, Daly MJ (2005): Haploview: analysis and visualization of LD and haplotype maps. *Bioinformatics*. 21:263-265.
4. Anthoni H, Zucchelli M, Matsson H, Muller-Myhsok B, Fransson I, Schumacher J, *et al.* (2007): A locus on 2p12 containing the co-regulated MRPL19 and C2ORF3 genes is associated to dyslexia. *Hum Mol Genet*. 16:667-677.
5. Meng H, Smith SD, Hager K, Held M, Liu J, Olson RK, *et al.* (2005): DCDC2 is associated with reading disability and modulates neuronal development in the brain. *Proc Natl Acad Sci U S A*. 102:17053-8.
6. Schumacher J, Anthoni H, Dahdouh F, Konig IR, Hillmer AM, Kluck N, *et al.* (2006): Strong genetic evidence of DCDC2 as a susceptibility gene for dyslexia. *Am J Hum Genet*. 78:52-62.
7. Harold D, Paracchini S, Scerri T, Dennis M, Cope N, Hill G, *et al.* (2006): Further evidence that the KIAA0319 gene confers susceptibility to developmental dyslexia. *Mol Psychiatry*. 11:1085-1091, 1061.
8. Cope N, Harold D, Hill G, Moskvina V, Stevenson J, Holmans P, *et al.* (2005): Strong evidence that KIAA0319 on chromosome 6p is a susceptibility gene for developmental dyslexia. *Am J Hum Genet*. 76:581-591.
9. Francks C, Paracchini S, Smith SD, Richardson AJ, Scerri TS, Cardon LR, *et al.* (2004): A 77-kilobase region of chromosome 6p22.2 is associated with dyslexia in families from the United Kingdom and from the United States. *Am J Hum Genet*. 75:1046-1058.
10. Dennis MY, Paracchini S, Scerri TS, Prokunina-Olsson L, Knight JC, Wade-Martins R, *et al.* (2009): A common variant associated with dyslexia reduces expression of the KIAA0319 gene. *PLoS Genet*. 5:e1000436.
11. Newbury DF, Winchester L, Addis L, Paracchini S, Buckingham LL, Clark A, *et al.* (2009): CMIP and ATP2C2 modulate phonological short-term memory in language impairment. *Am J Hum Genet*. 85:264-272.
